# Impact of Denture Prostheses on Cognitive Functioning in Completely Edentulous Patients: A Pilot Study

**DOI:** 10.7759/cureus.43570

**Published:** 2023-08-16

**Authors:** Syed Ershad Ahmed, Ramesh Raju, Anjana Kurien, Kanaha M, Sidra Bano, Hemcle Shalma

**Affiliations:** 1 Prosthodontics, Vinayaka Missions Research Foundation (Deemed to be University), Salem, IND; 2 Prosthodontics and Crown & Bridge, Sri Ramakrishna Dental College and Hospital, Coimbatore, IND; 3 Prosthodontics, Vinayaka Missions Sankarachariyar Dental College, Vinayaka Missions Research Foundation (Deemed to be University), Salem, IND; 4 Prosthodontics, Sri Ramakrishna Dental College and Hospital, Coimbatore, IND; 5 Pharmacology, Ultra Best Dental College and Hospital, Madurai, IND; 6 Oral Medicine and Radiology, Sri Ramakrishna Dental College and Hospital, Coimbatore, IND

**Keywords:** geriatric, completely edentulous, cognitive functioning, alzheimer's disease, dementia, denture

## Abstract

Aim: The aim is to evaluate the impact of denture prostheses on cognitive functioning in completely edentulous patients using a novel cognitive assessment tool.

Methods: Thirty (n=30) completely edentulous patients of age groups above 60 years were taken in the present study group. Pre- and post-cognitive assessments were done on the patients using a novel cognitive assessment tool, Dental Cognitive Functioning Assessment Tool (DCFAT). These assessments were done in the pre-treatment stage and after two weeks and three months of prostheses function.

Results: The mean DCFAT score of 30 patients shows 10.13 recorded prior to denture fabrication and 11.5 and 14 after two weeks and three months of prosthesis function, respectively. A mean difference in the DCFAT score of 1.37 was seen between the pre-denture fabrication stage and two weeks of prosthesis function. The mean difference in DCFAT score of 3.87 was seen between the pre-denture fabrication stage and three months of prosthesis function and the mean difference score of DCFAT score 2.5 was observed after two weeks and three months of prosthesis function. One-way ANOVA was used to investigate the statistical difference between bivariate samples followed by the post hoc Tukey test. The results were statistically significant p < 0.00001.

Conclusion: The inference obtained suggest that the replacement of missing teeth by denture prostheses enhances the cognitive functioning in the elderly population which can eventually reduce the occurrence of dementia.

## Introduction

Dementia and Alzheimer’s related problems are common in the elderly aged population leading to a decline in cognitive thinking and functioning. Masticatory efficiency of an individual is associated with the dentition present in the oral cavity and teeth loss due to any reason results in decreased neurodegenerative cognitive function [1]. It has been suggested that chewing efficiency is allied with the activity of the cerebral cortex and also the function of chewing increases the flow of blood in the cerebral region, thereby increasing oxygen levels in the pre-frontal and hippocampus area [2]. Thus, masticatory insufficiency tends to bring a gradual decline in cognitive functioning. Therefore, it is recommended in older individuals replace their missing teeth so that their chewing efficiency is increased [2]. Rehabilitation by dental prostheses either removable dentures or through fixed prostheses aids in the modeling of cortices related to the chewing or mastication situated in the prefrontal and parietal sensorimotor areas, thus improving the individual's cognitive capacity and also reducing the possibilities of dementia or Alzheimer-related problems [3-5].

Numerous cognitive screening and assessment tools are available to assess the cognitive functioning status of an individual, to name A few are the Mini-mental state examination (MMSE), Montreal cognitive assessment (MOCA) General practitioner assessment of cognition (GPCOG) Mini-cog evaluation, and Dementia assessment by rapid test (DART). All these screening and assessment tools use parameters such as orientation, naming, memory, language, and visuospatial thinking to assess cognitive functioning. 

Removable denture prostheses and implant‑supported prostheses tend to improve the individual's nutritional status and improve the perception of oral health in the geriatric population, thereby enhancing their cognitive capacity [5,6]. Hence, dental professionals play a major role in the oral rehabilitation of missing teeth, thus demanding a novel cognitive assessment tool specially designed for dental professionals which is easy to use and also incorporates dental terms in assessing the cognitive capacity of an individual.

Thus, from the above introduction, the present aim of the study was to evaluate the impact of denture prostheses on cognitive functioning in completely edentulous patients using a novel cognitive assessment tool namely the Dental Cognitive Functioning Assessment tool (DCFAT).

Objectives

The objective is to assess the cognitive functioning score of the completely edentulous patients before and after the fabrication of the prostheses, thereby assessing whether removable denture prostheses have enhanced cognitive functioning.

## Materials and methods

The study protocol and informed consent form were approved by the ethical committee of the Vinayaka Mission’s Research Foundation, Salem, Tamil Nadu, India. Thirty (n=30) completely edentulous patients of the age group above 60 years were taken in the present study group. Patients with neuromuscular, and neurological problems and any disorders of GIT/systemic diseases with nutritional deficiencies related to missing teeth were excluded from the study. The sampling design used in the study was Systematic random sampling. All the patients were rehabilitated with complete denture prostheses and informed consent was obtained from all participating patients. The study was conducted in Vinayaka Missions Sankarachariyar Dental College and Hospital, Salem, and in Sri Ramakrishna Dental College and Hospital, Coimbatore.

To assess the cognitive functioning score levels of the patients a novel assessment tool known as DCFAT was developed. The validity and reliability were tested by the content validity tool. The internal consistency of the questions was assessed by Alpha Cronbach’s Test.

**Figure 1:**
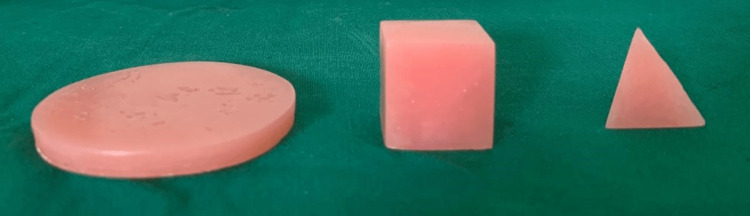
Shapes of acrylic blocks used in the cognitive functioning evaluation tool

DCFAT and the subscales are calculated by summing the score of the responses to 6 items and items corresponding to the subscales. One of the subscales included the naming of an object corresponding to a shape such as a square, rectangle, or triangle made of heat cure acrylic resin (Figure 1). The shape specimens were disinfected with 0.5% of Sodium Hypochlorite for 10 minutes (immersion technique). Later, the specimens were washed with water and dried before placing them in the oral cavity [7].

Total DCFAT scores range from 1 to 15 (Appendix). The scales suggest that a higher score shows significant improvement in the cognitive functioning of the patient with denture prostheses in terms of their orientation, naming, language (speech), oral stereognosis, proprioception, and memory. The tool was assessed in the pre-treatment stage and subsequently after two weeks and three months of prosthesis function for all rehabilitated patients.

SPSS statistics software 23.0 version (IBM Corp., Armonk, NY, USA) was used to analyze the collected data. Mean and SD were calculated for continuous variables. One-way ANOVA was used to investigate the statistical difference between bivariate samples followed by the post hoc Tukey test. The P-value was assigned to be p < 0.05.

## Results

The DCFAT was assessed in all the 30 patients. The six sub scales were assessed individually prior to denture fabrication and after prostheses functioning. Later scores were assigned and mean and standard deviation were calculated for each subscale (Table 1).

**Table 1:** Individual Dental Cognitive Functioning Assessment Tool (DCFAT) score of six subscales

	1. Orientation			2. Language (speech)			3. Naming			4. Oral stereognosis			5. Memory			6. Proprioception		
N = 30	Pre-treatment	Post-treatment 2 weeks	Post-treatment 3 months	Pre-treatment	Post-treatment 2 weeks	Post-treatment 3 months	Pre-treatment	Post-treatment 2 weeks	Post-treatment 3 months	Pre-treatment	Post-treatment 2 weeks	Post-treatment 3 months	Pre-treatment	Post-treatment 2 weeks	Post-treatment 3 months	Pre-treatment	Post-treatment 2 weeks	Post-treatment 3 months
1	3	3	4	1	2	2	3	3	3	1	1	2	1	1	2	1	1	1
2	3	3	4	1	2	2	3	3	3	1	1	2	1	1	2	0	1	1
3	5	5	5	2	2	3	2	2	2	2	2	2	2	2	2	0	0	1
4	2	2	4	1	1	2	3	3	3	1	1	2	1	2	2	0	0	0
5	3	3	4	2	1	3	3	3	3	2	2	2	2	2	2	1	1	1
6	4	4	5	1	2	2	3	3	3	0	1	2	1	1	1	0	1	1
7	3	3	4	1	1	2	3	3	3	0	1	2	1	2	2	1	1	1
8	3	3	5	1	2	2	3	3	3	0	1	2	1	2	2	0	0	0
9	4	4	4	2	1	2	3	3	3	1	1	2	1	2	2	0	0	0
10	3	3	4	1	1	2	3	3	3	1	2	2	1	2	2	1	1	1
11	2	2	4	2	2	2	3	3	3	0	1	1	1	2	2	1	1	1
12	3	3	3	1	1	2	3	3	3	1	1	2	1	2	2	0	1	0
13	3	3	3	1	1	2	2	2	2	1	2	2	1	1	2	0	1	1
14	5	5	5	2	2	3	3	3	3	2	2	2	2	2	2	0	0	1
15	4	4	4	2	2	3	3	3	3	1	1	2	1	1	1	1	1	1
16	3	3	3	2	2	3	3	3	3	2	2	2	2	2	2	0	1	1
17	4	4	4	1	1	2	3	3	3	0	1	1	1	1	2	1	1	1
18	3	3	4	2	2	3	3	3	3	0	1	1	1	1	2	0	0	1
19	3	3	3	1	1	2	3	3	3	0	1	1	1	2	2	0	0	1
20	3	3	4	2	2	2	3	3	3	1	2	2	1	2	2	1	1	1
21	3	3	5	1	1	2	3	3	3	1	1	2	1	2	2	0	1	1
22	3	3	5	2	2	3	3	3	3	1	2	2	1	1	2	0	1	1
23	4	4	4	1	1	2	3	3	3	1	1	2	1	2	2	0	0	0
24	3	3	5	2	2	3	3	3	3	1	1	2	1	2	2	1	1	1
25	4	4	4	1	1	2	3	3	3	2	2	2	2	2	2	0	0	1
26	4	4	4	2	2	3	3	3	3	1	1	2	1	1	1	1	1	1
27	3	3	4	1	2	2	3	3	3	2	2	2	2	2	2	0	1	1
28	4	4	5	1	1	2	2	3	3	0	1	2	1	2	2	0	1	1
29	3	3	5	1	1	2	3	3	3	0	1	2	1	2	2	1	1	1
30	4	4	4	1	1	2	3	3	3	0	1	2	1	1	2	1	1	1
Mean	3.39	3.39	4.17	1.42	1.46	2.32	3	3	3	0.85	1.35	1.86	1.2	1.66	1.9	0.4	0.7	1
SD	0.71	0.71	0.64	0.49	0.50	0.466	0.30	0.25	0.25	0.73	0.47	0.34	0.40	0.47	0.30	0.49	0.46	0.37

With respect to the dental cognitive functional assessment, the mean scores of five subscales such as orientation (Mean = 3.39,3.39,4.17), language (Mean = 1.42,1.46,2.32), oral stereognosis (Mean = 0.85,1.35,1.86), memory (Mean = 1.2,1.66,1.9) and proprioception (Mean = 0.4,0.7,1.0) were found to have a significant improvement from pre-treatment stage to the post-treatment stage (prostheses functioning after two weeks and three months). One among subscales in the tool Naming (Mean= 3,3,3) had not much in variation during the pre-treatment stage to the post-treatment stage as most of the patients were able to identify and name the shapes showed in the assessment tool.

Mean DCFAT score of 30 patients was 10.13 recorded prior to denture fabrication and 11.5 and 14 after two weeks and three months of prosthesis function, respectively (Table 2).

**Table 2:** Mean and SD of Dental Cognitive Functioning Assessment Tool (DCFAT) - pre-treatment and two weeks and three months of denture prostheses function

S. No	Pre-treatment	Post-treatment, 2 weeks	Post-treatment, 3 months
1	10	11	14
2	9	11	14
3	13	13	15
4	8	9	13
5	13	12	15
6	9	12	14
7	9	11	14
8	8	11	14
9	11	11	13
10	10	12	14
11	9	11	13
12	9	11	12
13	8	10	12
14	14	14	16
15	12	12	14
16	12	13	14
17	10	11	13
18	9	10	14
19	8	10	12
20	11	13	14
21	9	11	15
22	10	12	16
23	10	11	13
24	11	12	16
25	12	12	14
26	12	12	14
27	11	13	14
28	8	12	15
29	9	11	15
30	10	11	14
MEAN	10.13	11.5	14
SD	1.67	1.074	1.082

Mean difference in DCFAT score of 1.37 was seen between pre-denture fabrication stage and two weeks of prosthesis function. An increase of 12.6% was seen in the cognitive functioning score between the pre-treatment stage and two weeks of prosthesis function.

Mean difference score of DCFAT 2.5 was observed after two weeks and three months of prosthesis function. A total of 19.6% increase in the cognitive functioning score was noted between the two evaluations (two weeks and three months of prosthesis function).

**Figure 2:**
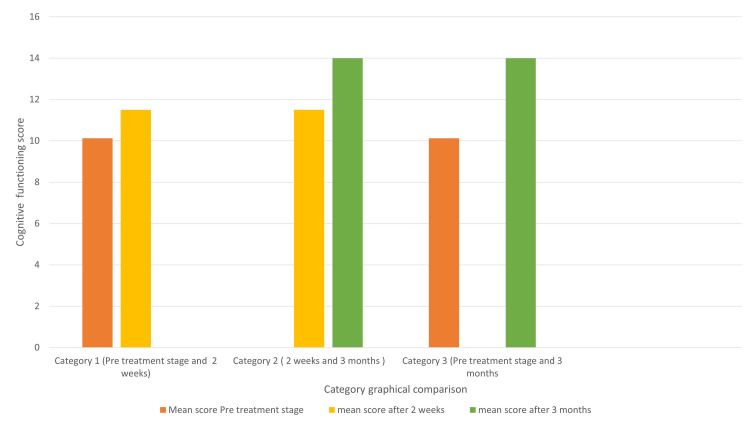
Mean score among three evaluation criteria (pre-treatment, after two weeks of prostheses functioning, and after three months of prostheses functioning)

Mean difference in DCFAT score of 3.87 was seen between pre- denture fabrication stage and three months of prosthesis function. 32% of increase was noted in cognitive functioning score among the patients after three months of prostheses functioning when compared to their pre-treatment stage values (Figure 2).

One-way ANOVA analysis was used to investigate the statistical difference between bivariate samples followed by post hoc Tukey test. P-value was assigned to be <0.05. When analyzing the DCFAT scores statistically significant results were observed p < 0.00001 (Table 3).

**Table 3:** One-way ANOVA treatment analysis

	1	2	3	Total
N	30	30	30	90
∑X	304	345	420	1069
Mean	10.1333	11.5	14	11.878
∑X^2^	3,162	4,001	5,914	13,077
Std. Dev.	1.6761	1.0748	1.0828	2.065
Std Error	0.306	0.1962	0.1977	

After the ANOVA treatment, f-ratio value was determined (Table 4).

**Table 4:** One-way ANOVA analysis result details The f-ratio value is 67.36384. The p-value is < 0.00001. The result is significant at p < 0.05.

Source	Sum of squares (SS)	Degree of freedom (df)	Mean square (MS)
Between-treatments	230.689	2	115.3444
Within-treatments	148.967	87	1.7123
Total	379.656	89	

One-way ANOVA analysis was followed by post -hoc Tukey test (Table 5).

**Table 5:** Post-hoc Tukey test analysis

Pairwise comparisons		HSD_.05_ = 0.8056	Q_.05_ = 3.3722, Q_.01_ = 4.2308
		HSD_.01_ = 1.0108	
T_1_:T_2_	M_1_ = 10.13	1.37	Q = 5.72 (p = 0.00033)
	M_2_ = 11.50		
T_1_:T_3_	M_1_ = 10.13	3.87	Q = 16.19 (p = 0.00000)
	M_3_ = 14.00		
T_2_:T_3_	M_2_ = 11.50	2.5	Q = 10.46 (p = 0.00000)
	M_3_ = 14.00		

## Discussion

A drastic rise in the elderly population is expected in India by 2050, due to increased life expectancy caused by recent technologies in the medical field. Studies also suggest that dementia cases are expected to increase rapidly by 2030 [8].

Seraj et al. concluded in their study that elderly people having good chewing efficiency had enhanced cognitive capacity and memory recovery. From the study, the author found that individuals with fewer dentitions had low MMSE scores leading to decreased cognitive function [9].

Narita et al. [10] mentioned in the study that removable partial denture prostheses led to enhanced dorsal prefrontal cortex activation. The results of the author emphasized that partial denture prostheses activate the masticatory muscles and also help in preventing cognitive decline. The results obtained in the above study were similar to the results in our study, there was an improved masticatory efficiency among the individuals from the pretreatment stage to the post-evaluation stage.

There was a 12.6% increase in the DCFAT score seen in the patients prior to denture fabrication (pre-treatment stage) and after two weeks of prostheses function. This increased cognitive score can be related to the findings suggested by Reibeiro et al. that new prostheses have an increased number of teeth and more surface area for mastication and thereby increasing chewing efficiency leading to higher mental examination scores [11].

Comparing the DCFAT score of the pre-treatment stage and three months of prostheses function, the percentage increase in cognitive score was around 32%. This high percentage can be attributed to the good functional quality of the denture which was fabricated for the patients as the quality of the denture plays an important role in maintaining the muscle tissue and preserving the bone, which eventually helps in increased chewing efficiency [12].

There was an increase of 19.6% in the DCFAT score in the patients after two weeks and three months of prostheses function. This increase in the score shows the transition phase of denture adaptation and acceptance in the patient, as better retentive and fit prostheses with good occlusal contacts can enhance the chewing efficiency of the patients leading to a good cognitive examination score [13].

From the above data obtained in the study, it is understood that mastication is important in directly influencing the cognitive capacity of an individual, and mastication is achieved through proper occlusal contacts and having an increased number of occlusal units. Through oral stereognosis perception, the patient can identify the consistency and texture of the food placed in the mouth. Denture fabricated with proper occlusal units and contacts will increase this perception capacity and thus the oral stereognosis sub-scale has been gradually increased among patients rehabilitated with complete dentures in our study. The results obtained in the study correlate with the findings obtained in the studies suggested by Kim et al. [14], Campos et al. [13], Cerruti et al. [12], and Reibeiro et al. [11].

Statically significant differences (p < 0.00001) were observed among pre-treatment and two weeks, and three months of prostheses function in the cognitive functioning assessment scale. In this study, we also concluded that there was a positive impact of denture prostheses on oral health and quality of life among completely edentulous patients.

As the study was a pilot study the number of samples was limited to thirty patients, therefore an increased number of samples with a much longer evaluation period (six months and one year after the denture prosthesis's function) can be employed in future studies to learn the effects of denture prostheses on the cognitive functioning and also in future the results obtained with the new cognitive assessment tool should be assessed with an another established cognitive assessment tool, like MOCA, etc., to know whether the results are in concordance with the established tool. So, within the limitations of this study, it can be concluded that there exists a highly positive correlation between denture prostheses and cognitive functioning in completely edentulous patients.

## Conclusions

The inference obtained from the study suggests that the replacement of missing teeth by conventional removable complete denture prostheses increases the masticatory efficiency in the subjects, thereby enhancing the cognitive functioning of the subjects seen between the pre-treatment stages to three months of prostheses functioning. Therefore, to reduce cognitive decline among the completely edentulous patients, dentists and oral health specialists should play an active role and motivate people in oral rehabilitation.

## Appendices

**Table 6:** Dental Cognitive Functioning Assessment Tool (DCFAT) – novel cognitive assessment tool with dental parameters Overall assessment score (max points – 16); normal – between 10 and 15

S.no	Domain/Item	Description	Scoring pattern	Score
1.	ORIENTATION	Date -	1 point for each	
		Month -		
		Year -		
		Day -		
		Place -		
		2.			LANGUAGE (SPEECH)	a) Speak certain words with letter M and F in 1 minute.	1 point for each (if educated add 1 point )	
b) Asking the patient to speak and repeat sentences in their native language								
c) Read certain words (if educated)								
3.	NAMING	Identify the shape and name them individually	1 point for each shape	
4.	ORAL STEREOGNOSIS	a) Identify the shape of the object placed in the mouth	1 point for each	
		b) Draw the shape of the object		
5.	MEMORY	a) Read the words, Repeat the words for three times Recall the words after 5 minutes Words: (if educated) Mississippi Face Fan Any colour Any animal or if the individual knows only the native language, words can be of native language	1 point for each	
		b) recall the shape of the objects placed in the mouth in step 4		
6.	PROPRIOCEPTIVE MOVEMENTS	Ask the individual to perform the movements of the mandible Protrusive	1 point for the entire question	
		Right lateral		
		Left lateral		
		Opening		
		Closing		

## References

[1] Kamiya K, Narita N, Iwaki S (2016). Improved prefrontal activity and chewing performance as function of wearing denture in partially edentulous elderly individuals: functional near-infrared spectroscopy study. PLoS One.

[2] Ki S, Yun J, Kim J, Lee Y (2019). Association between dental implants and cognitive function in community-dwelling older adults in Korea. J Prev Med Public Health.

[3] Chuhuaicura P, Dias FJ, Arias A, Lezcano MF, Fuentes R (2019). Mastication as a protective factor of the cognitive decline in adults: a qualitative systematic review. Int Dent J.

[4] Klotz AL, Hassel AJ, Schröder J, Rammelsberg P, Zenthöfer A (2017). Oral health-related quality of life and prosthetic status of nursing home residents with or without dementia. Clin Interv Aging.

[5] Ahmed SE, Mohan J, Kalaignan P, Kandasamy S, Raju R, Champakesan B (2021). Influence of dental prostheses on cognitive functioning in elderly population: a systematic review. J Pharm Bioallied Sci.

[6] El Osta N, El Osta L, Moukaddem F, Papazian T, Saad R, Hennequin M, Khabbaz LR (2017). Impact of implant-supported prostheses on nutritional status and oral health perception in edentulous patients. Clin Nutr ESPEN.

[7] Chau VB, Saunders TR, Pimsler M, Elfring DR (1995). In-depth disinfection of acrylic resins. J Prosthet Dent.

[8] Sathianathan R, Kantipudi SJ (2018). The dementia epidemic: Impact, prevention, and challenges for India. Indian J Psychiatry.

[9] Seraj Z, Al-Najjar D, Akl M, Aladle N, Altijani Y, Zaki A, Al Kawas S (2017). The effect of number of teeth and chewing ability on cognitive function of elderly in UAE: a pilot study. Int J Dent.

[10] Narita N, Kamiya K, Yamamura K, Kawasaki S, Matsumoto T, Tanaka N (2009). Chewing-related prefrontal cortex activation while wearing partial denture prosthesis: pilot study. J Prosthodont Res.

[11] Ribeiro GR, Campos CH, Rodrigues Garcia RC (2017). Influence of a removable prosthesis on oral health-related quality of life and mastication in elders with Parkinson disease. J Prosthet Dent.

[12] Cerutti-Kopplin D, Emami E, Hilgert JB, Hugo FN, Padilha DM (2015). Cognitive status of edentate elders wearing complete denture: does quality of denture matter?. J Dent.

[13] Campos CH, Ribeiro GR, Costa JL, Rodrigues Garcia RC (2017). Correlation of cognitive and masticatory function in Alzheimer's disease. Clin Oral Investig.

[14] Kim MS, Oh B, Yoo JW, Han DH (2020). The association between mastication and mild cognitive impairment in Korean adults. Medicine (Baltimore).

